# Echocardiography in congenital mitral valve regurgitation–the liaison between cardiologist and surgeon

**Published:** 2009-11-25

**Authors:** C Ginghină, A Vlădaia, I Ghiorghiu, M şerban, BA Popescu, R Jurcuţ

## Abstract

Congenital heart diseases are broadly defined as those cardiac anomalies that are present at birth. By their very nature, such defects have their origin in embryonic development. Congenital mitral valve regurgitation is a rare disease occurring in infancy or childhood. In up to 60% of cases, congenital anomalies of the mitral valve occur in association with other cardiac lesions, and often more than one component of the mitral apparatus is involved. The true incidence of congenital mitral valve regurgitation (MVR) is difficult to determine accurately (0.21–0.42% from total mitral valve regurgitations); isolated congenital mitral regurgitation is uncommon.

The Carpentier classification of congenital mitral valve disease is the most commonly used nomenclature based on a functional analysis of the mitral valve leaflet. The contemporary anatomic classification has the advantage of minimizing observer variability in the diagnosis and it offers a much better liaison between the cardiologist and surgeon.

Historically, the emergence of two–dimensional echocardiography must be viewed as a milestone in the diagnostic approach to congenital heart disease. The tomographic nature of the technique and its great number of imaging planes permit the anatomy and relationships of the cardiac structures to be defined, even in the presence of complex congenital malformations. For the noninvasive assessment of cardiac structure and function, echocardiography plays a prominent role as the most accurate and widely applied method. When details of the clinical history are unavailable, the echocardiographer is often called on to determine which surgical procedures have been performed. The options for further intervention often depend on the echocardiographic results [1,2].

For a good and useful evaluation, echocardiographic exam must take into account the anatomic and functional aspects of mitral valve, in a tight connection. To obtain a comprehensive and accurate description of the whole mitral apparatus it is useful to have a systemic approach when performing transthoracic echocardiography and multiplane transesophageal echocardiography examination.

The anatomic structure of mitral apparatus is complex and sometimes difficult to describe in echocardiographic exam [3].

Anatomically, the mitral valve consists of an annulus, leaflets, chordae tendineae, and papillary muscles. The mitral annulus is an integral part of the fibrous skeleton of the heart. The normal mitral valve has two leaflets, anterior and posterior, that act in conjunction with the subvalvular apparatus as one functional unit. The larger anterior (i.e., septal or aortic) leaflet attaches to 150 degrees of the annulus, and is squat and trapezoidal in shape. As a consequence of being in fibrous continuity with the aortic valve, it forms the posterior boundary of the left ventricular outflow tract. The posterior (i.e., mural) leaflet is narrower and occupies 210 degrees of the annulus. Two natural indentations in the posterior leaflet produce three segments, which subdivide it into lateral, central and medial scallops. The mitral valve leaflets are separated by the anterolateral and posteromedial commissures. Beneath the commissures lie two corresponding papillary muscles, which are extensions of the subendocardial ventricular myocardium. Chordae tendineae from the papillary muscles insert on both sides of the corresponding commissure, so each valve leaflet receives chordae from both papillary muscles.

Considerable variation can be found in the morphology of the normal mitral valve [4, 5]. It is now widely recognized that the function of the mitral valve depends on the normal function and integrity of the leaflets, annulus, chordae tendineae, papillary muscles, and subjacent left ventricular myocardium. Abnormalities in any of these components, either individually or in combination, produce dysfunction of the mitral valve unit.

It should be expected that medical and surgical practice use prevalently an anatomic classification. However, the Carpentier classification [6] of congenital mitral valve disease (Table 1) is the most commonly utilized nomenclature both in medical and surgical practice

**Table 1:** Classification of congenital mitral valve regurgitation according to the Carpentier functional approach [3,6,7]

Mitral valve incompetence
Type Ⅰ (normal leaflet motion)	Type Ⅱ (leaflet prolapse)	Type Ⅲ (restricted leaflet motion)
Annular dilatation Cleft anterior leaflet Leaflet defect–partial leaflet agenesis	Chordal elongation Papillary muscle elongation Chordal agenesis	Type ⅢA (normal papillary muscles) papillary muscle commissural fusion shortened chordae excessive leaflet tissue valvar ring annular hypoplasia	Type ⅢB (abnormal papillary muscles) parachute mitral valve papillary muscle hypoplasia hammock mitral valve

This 1976 classification is predicated on a functional analysis of the mitral valve leaflet. However, there are two limitations of this classification, which can produce observer variability, and therefore potential inconsistency in data reporting. Firstly, a purely stenotic or insufficient valvar lesion is rarely observed. Secondly, the surgical literature has generally divided, reported, and risk stratified congenital mitral valve lesions based on stenosis versus insufficiency, not leaflet function [4].

In addition, the Carpentier classification was developed before routine two–dimensional echocardiography was available. Functional evaluation of the leaflets by direct vision in the operating room may be difficult, despite saline insufflation of the flaccid left ventricle [4].

Accurate and thorough preoperative echocardiography becomes mandatory to define lesions based on leaflet motion and to effect the appropriate repair. Individual interpretations of the functional anatomy introduce variability, which makes the reliability and reproducibility of a classification difficult [4, 8, 9].

At present, transesophageal echocardiography provides considerable information regarding mitral valve structure and function. Intraoperative echocardiographic monitoring of mitral valve surgery has become a routine practice in most cardiac centers. Perioperative assessment of valve anatomy by transesophageal approach provides detailed anatomical imaging [9].

Surgeons now have much more information before the valve is inspected. This permits preoperative classification, which may be enhanced by direct observation of the valve at the time of operation.

Within the context of criticisms for Carpentier functional classification of congenital mitral valve diseases and from practical necessity, S. N. Mitruka et al. [4] (Table 2) proposed a contemporary unifying anatomic classification for congenital abnormalities of the mitral valve based on a consideration of whether the valve is stenotic or insufficient.

The adoption of an optimal standard nomenclature that could be widely utilized, consistent, and reproducible would minimize observer variability in the diagnosis. This classification makes the issues of leaflet function as a consequence of the altered valvar anatomy. This, in turn, allows a segmental and systematic approach for considering the therapeutic options [10, 11, 15].

The anatomic contemporary classification for congenital mitral valve disease is presented below (Table 2).

In our institution (‘Prof. C.C. Iliescu’ Emergency Institute of Cardiovascular Diseases) from March 2003 to March 2009 were admitted 60.175 patients, out of whom 8709 (14,47%) had mitral valve regurgitation. Congenital mitral valve regurgitation was represented by 4.08% (356 patients): 197 men and 156 women, with medium age of 48.3 years old (range: 5–89 yrs). We have to add that in our Institute, which includes a Medical Cardiology Clinic and a Department of Cardiac Surgery, Carpentier classification is used dominantly, and the prevalent surgical treatment is mitral valve replacement. However, lately, the anatomic classification has been starting to gain its place. Thus, there were cases of mitral valve disease in which anatomic classification offered a comprehensive medical and surgical approach.

**Table 2:** Contemporary classification for congenital mitral valve regurgitation [4, 10, 11]

Congenital mitral valve regurgitation
Type 1	Type 2	Type 3	Type 4
Supravalvar	Valvar	Subvalvar	Mixt
Mitral ring circumferential ridge of endocardial tissue the underlying valve is abnormal (stenotic or hipoplastic) may be associated with stenotic lesions (parachute or hammock valve; papillary muscle fusion; double orifice mitral valve) may induce insufficiency differentiated from cor triatriatum	Annulus midvalvar ring (obstructive lession associated) hypoplasia (associated with hypoplastic left ventricles, ventricular septal defect and aortic coarctation) dilatation (associated with secundum atrial septal defect) deformation Leaflet hypoplasia/agenesis cleft excessive tissue double orifice mitral valve	Chordae tendineae agenesis (leaflet prolapse) hortened–funnel valve (leaflet limited mobility) elongated (leaflet prolapse) Papillary muscles hypoplasia/agenesis(valvar incompetence) shortened (valvar incom–petence and mitral stenosis) single–parachute valve (valvar insufficiency: cleft leaflet, poorly developed anterior leaflet, short chordae, annular dilatation) multiple–hammock valve (valvar insufficiency: cleft leaflet, anterior leaflet hypoplasia, shortened chordae, annular dilatation)	Combination of two or more of the others three types

In virtue of this classification, we present some personal echocardiographic images with congenital mitral valve regurgitation, as it follows:

- **Type 1 (supravalvar)**, an abnormality of the mitral valve above the level of the leaflets or annulus, is illustrated in**Fig 1** by a mitral supravalvular ring (a circumferential ridge of endocardial tissue attached to the anterior leaflet below its insertion on the annulus).
- **Type 2 (valvar)**, an abnormality of the mitral valve at the level of leaflets or annulus, is illustrated in: **Fig 2** by isolated  mitral valve cleft,**Fig 3** by leaflets with excessive tissue and **Fig 4** by congenital double–orifice mitral valve.
- **Type 3 (subvalvar)**, an abnormality of the mitral valve at the level of the chordae tendinae or the papillary muscles, is illustrated in **Fig 5** by elongated chordae tendineae and in **Fig 6** by shorted chordae tendineae.
- **Type 4 (mixed)**, an abnormality of the mitral valve that is described as a mixture or combination of two or more of the above three types, described in **Fig 7** by: annulus dilatation, excessive tissue on the leaflets and elongated chordae tendineae

**Fig 1:**
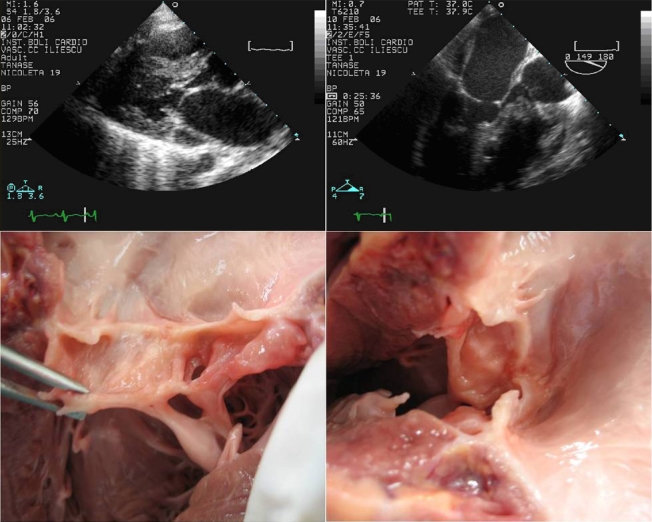
**Transthoracic echocardiography**: parasternal long axis modified view (A) shows a circumferential ridge of endocardial tissue attached to the anterior leaflet below its insertion on the annulus and to the atrium (arrows); respectively, transesophageal echocardiogram long–axis view of the left ventricle (B) showing the same supravalvar membrane (arrow) and anatomic pieces (C, D) with the mitral ring from left atrium sight (arrows).

**Fig 2:**
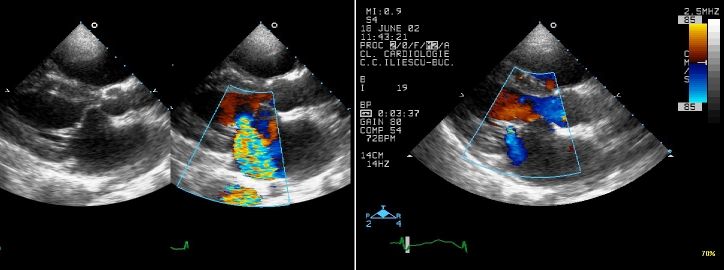
**Two D (2D) Transthoracic echocardiography**: parasternal long axis view from a patient with isolated mitral valve cleft: note preoperative severe mitral valve regurgitation (on color flow Doppler: the regurgitant jet passes through the cleft of the anterior mitral valve leaflet, and it is directed posteriorly) (A); and postoperative, after repairing the cleft, a mild mitral valve regurgitation (B).

**Fig 3:**
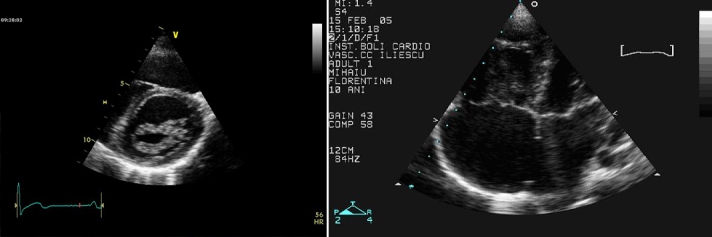
**Two (2D) Transthoracic echocardiography**: parasternal short axis view at the level of mitral valve (A) shows mitral valve leaflets with excessive tissue from a patient with mild mitral valve regurgitation and the apical four chambers view (B) shows secundum atrial septal defect (arrow) in the same patient.

**Fig 4:**
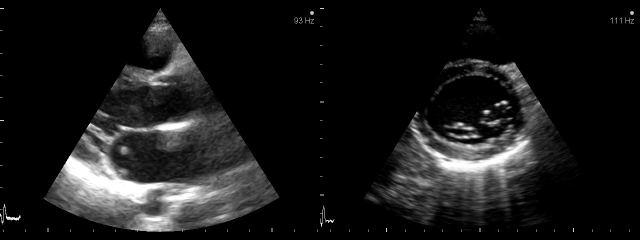
**Two (2D) Transthoracic echocardiography**: parasternal long axis view (A) shows large vegetations on the both mitral valve leaflets (white arrow), and parasternal short axis view at the level of mitral valve (B) which shows the mitral valve with two orifices opening into the left ventricle, from a 10 year– old girl with a severe mitral valve regurgitation. In this case we have to remark that although echocardiographic aspect was highly suggestive for congenital double–orifice mitral

**Fig 5:**
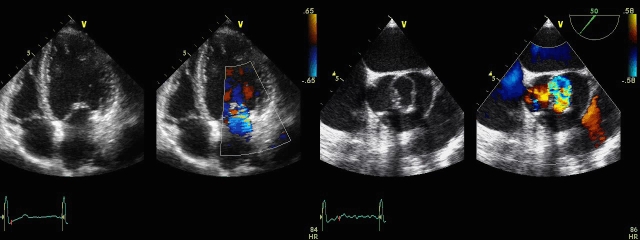
**Two (2D) Transthoracic echocardiography**: apical four chamber view–2D and color flow Doppler (A) shows a mild mitral valve regurgitation on a valve with elongated chordae tendineae in a 24 year–old woman and parasternal short axis view–2D and color flow Doppler (B)  from the same patient, which shows associated  bicuspid aortic valve with moder

**Fig 6:**
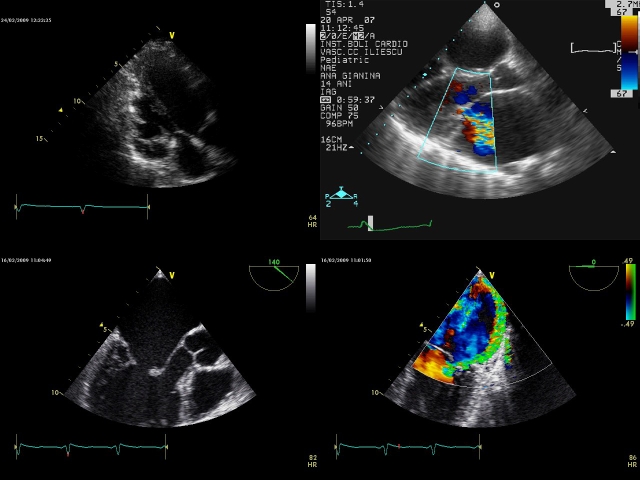
**Two (2D) Transthoracic echocardiography**: parasternal long axis modified view (A) and normal parasternal long axis view with color Doppler flow (B) from an adult patient with moderate mitral valve regurgitation on shortened chordae tendineae; respectively transesophageal echocardiograms: 2D (C) and color flow Doppler (D) from another adult patient with severe mitral valve regurgitation on shortened cordae tendineae (note: posterior mitral leaflet with limited mobility–arrow, and eccentric regurgitant jet orriented to posterior atrial wall)

**Fig 7:**
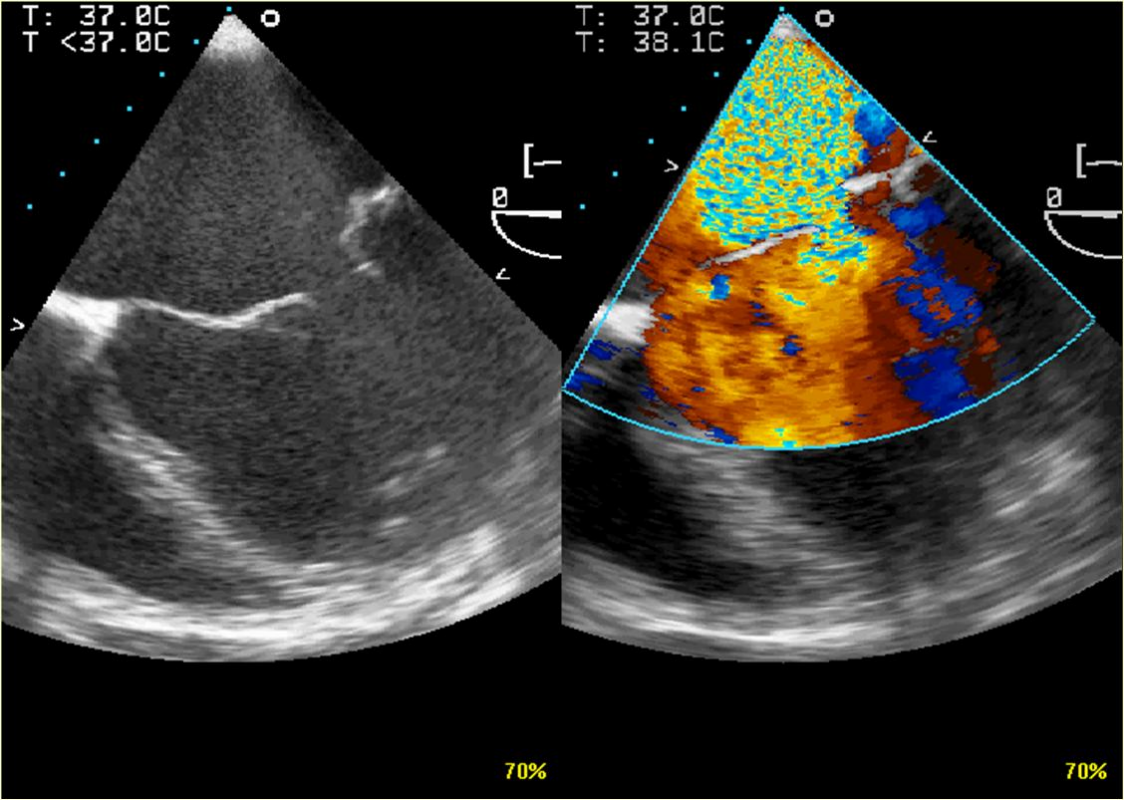
**Transesophageal echocardiogram**: 2D (A) and color Doppler flow (B) from a 27 year–old man, with Marfan syndrome. Note severe mitral valve regurgitation from: annulus dilatation, lengthening of the chordae tendineae and a redundancy of the leaflets, especially of the posterior one.

The therapeutic options for congenital mitral valve disease are somewhat limited [12, 13]. An operation on the mitral valve requires excellent exposure and considerable attention to detail.

The echocardiography can offer preoperative important features and allows the characterizations of the lesions based on both accepted classifications: the functional and the anatomical one. Currently, Doppler echocardiography plays an important role in the early determination of the type of surgery that may be performed for the correction of the mitral valve regurgitation [14– 16]. The morphological and functional aspects obtained on Doppler transthoracic and transesophageal echocardiography usually allow estimating with 85% the possibility of performing mitral valvuloplasty and its success in patients with myxomatous degeneration, particularly when the posterior leaflet is the most impaired one [17– 19].

There is no truly satisfactory substitute for the mitral valve in a patient of any age. The limitations of replacing the mitral valve with a mechanical valve in infants and children are well recognized [10]. Techniques for the repair of the mitral valve have become increasingly sophisticated in recent years [11] and may provide improvement in mitral valve function which permits delay in mitral valve replacement. It is possible that homograft replacement of the mitral valve will be a useful technique at some time in the future, but the technique must be considered investigational at the present time. 

Often, infants and children are referred for mitral valve operation in the absence of obvious symptoms. Gross cardiomegaly and dilatation of the left atrium in a seemingly asymptomatic child may provide an adequate basis for operation if the preoperative echocardiographic analysis suggests that the valve is amenable to repair. In patients with a markedly abnormal valve or in those who have previously undergone repair, mitral valve replacement may be the only option [20].

In general, most cardiologists and surgeons wait longer before referring a patient for mitral valve replacement than they would for a first time attempt at mitral valve repair. Thus, surgical intervention is considered when symptoms become severe or when exercise limitations become unacceptable. Valve repair is the optimal course of action. Mitral valve replacement is considered a last resort [21].

## Conclusion

The classification for congenital mitral valve regurgitation based dominantly on anatomic considerations offers useful medical and surgical details for the most appropriate mitral valve correction (valve repair being the optimal one). The Carpentier classification based on functional analysis of mitral valve leaflet continues to be the traditional classification, and may be used in addition to the anatomic nomenclature.
